# First computational characterization of *HTR5A-AS1*: a schizophrenia-linked antisense RNA with synaptic functions

**DOI:** 10.3389/fnins.2026.1716081

**Published:** 2026-01-27

**Authors:** Grant H. Ruttenberg

**Affiliations:** 1 Independent Researcher, Los Angeles, CA, United States; 2 Department of Surgery, David Geffen School of Medicine, University of California, Los Angeles (UCLA), Los Angeles, CA, United States

**Keywords:** *HTR5A-AS1*, long noncoding RNA, neurodevelopment, neurogenomics, schizophrenia, serotonin receptor, synaptic signaling, transcriptome-wide association study

## Abstract

**Background/objectives:**

Schizophrenia is a highly heritable psychiatric disorder that affects approximately 1% of the global population. Genome-wide association studies (GWAS) have mapped most schizophrenia risk variants to noncoding regions, highlighting the role of regulatory processes and noncoding RNAs in schizophrenia pathology. Despite this, and schizophrenia's association with 5-hydroxytryptamine (serotonin) system dysfunction, *HTR5A-AS1*, a long noncoding RNA (lncRNA) antisense to the serotonin receptor (HTR, 5-hydroxytryptamine receptor) gene *HTR5A*, remains virtually unstudied. This study provides the first systematic characterization of *HTR5A-AS1*, validating its transcript structure and investigating its genetic associations, expression dynamics, developmental regulation, and potential synaptic and GABAergic functions in schizophrenia.

**Methods:**

Transcriptome-wide association study (TWAS) summary statistics were integrated with postmortem RNA sequencing (RNA-seq), BrainSpan developmental transcriptomes, UCSC Genome Browser annotations, and functional prediction tools. These complementary approaches enabled validation of the transcript's structure, quantification of regional and developmental expression, and assessment of potential molecular functions.

**Results:**

*HTR5A-AS1* showed significant TWAS associations with schizophrenia in the hippocampus and dorsolateral prefrontal cortex (dlPFC). In postmortem schizophrenia donor tissue, expression was significantly reduced in the hippocampus, with a non-significant but directionally similar decrease in the dlPFC; sex-stratified analyses revealed that hippocampal reductions were strongest in male donors. Parallel analyses showed modest hippocampal downregulation of the paired receptor gene *HTR5A*, again driven primarily by males. Developmental transcriptomes revealed region-specific developmental trajectories, with steep increases during adolescence, aligning with the age range of typical schizophrenia onset. *HTR5A-AS1* was strongly co-expressed with *HTR5A*, and functional predictions implicated involvement in synaptic and GABAergic signaling, consistent with cortico-hippocampal circuit disruption in schizophrenia.

**Conclusions:**

These findings provide the first evidence that *HTR5A-AS1* is a bona fide antisense transcript with developmental and synaptic roles that may contribute to schizophrenia risk. Future single-cell and functional perturbation studies are needed to test causality and define mechanisms of regulation.

## 1 Introduction

Schizophrenia is a severe psychiatric disorder that impacts approximately 0.3%–0.7% of people during their lifetime, corresponding to about 21 million individuals worldwide (World Health Organization, 2025). Age-adjusted prevalence has remained relatively stable, but incidence rates have risen modestly over recent decades, with more new diagnoses per year (Solmi et al., 2023). Schizophrenia is characterized by hallucinations, delusions, cognitive deficits, and social withdrawal (Hany and Rizvi, 2025). Environmental factors such as maternal stress and especially adolescent cannabis use may contribute to a person's likelihood of getting schizophrenia. However, twin, family, and genome-wide association studies (GWAS) firmly establish genetics as the central risk component, with genetic factors explaining approximately 80% (Marder and Cannon, 2019) of the risk for schizophrenia. GWAS have identified 287 genetic variants and 120 genes linked to schizophrenia. Interestingly, of the 287 genome-wide significant risk loci, only 106 are in protein-coding genes (Chick et al., 2025; Huo et al., 2019). These findings highlight the importance of regulatory processes and noncoding RNAs as a risk factor for schizophrenia.

In addition, schizophrenia is widely considered a neurodevelopmental disorder, with symptoms typically emerging in adolescence or early adulthood. Converging evidence suggests that genetic risk factors act, in part, by altering neurodevelopmental trajectories: large-scale transcriptomic studies show that schizophrenia-associated genes are preferentially expressed prenatally and during adolescence, when synaptic pruning and cortical maturation occur (Birnbaum and Weinberger, 2017). This framework emphasizes the importance of evaluating potential risk genes across developmental stages and brain regions.

Schizophrenia is consistently linked to structural and functional abnormalities across brain regions. Meta-analyses and longitudinal magnetic resonance imaging (MRI) studies reveal reduced gray matter volumes and altered connectivity in areas including the prefrontal cortex, hippocampus, superior temporal cortex, thalamus, and anterior cingulate cortex (Karlsgodt et al., 2010). These structures are critical for cognition, memory, and emotional regulation, and their dysfunction highlights schizophrenia's characterization as a dysconnection syndrome, defined by disrupted cognition and impaired high-order neural integration.

The 5-hydroxytryptamine (serotonin) system has been consistently implicated in the pathology of schizophrenia, especially through its involvement in mood, cognition, and sensory processing (Quednow et al., 2020). The serotonin receptor family (HTR, 5-hydroxytryptamine receptor) consists of seven main classes from *HTR1A* to *HTR7* and 14 subtypes. Antipsychotic drugs used to treat schizophrenia primarily target dopamine receptors (Li et al., 2016), although many have also been shown to act on serotonin receptors (Meltzer et al., 2003), particularly *HTR1A* and *HTR2A*. Interestingly, variants in *HTR2A, HTR1A, HTR2C*, and *HTR3A* have been implicated in schizophrenia (Grubor et al., 2020).

By contrast, *HTR5A* is far less studied despite its clear physiological relevance. *HTR5A* encodes the serotonin 5-HT5A receptor, a G protein-coupled receptor (GPCR) that inhibits adenylate cyclase and modulates cyclic adenosine monophosphate (cAMP) signaling (Thomas, 2006). *HTR5A* is highly expressed in the cortex and hippocampus (Thomas, 2006), areas that are consistently disrupted in schizophrenia. Nevertheless, unlike *HTR2A* and *HTR1A*, which are highly characterized, *HTR5A*'s role in psychiatric disease remains largely unexplored. This gap makes it especially intriguing to investigate *HTR5A* as a potential contributor to the pathophysiology of schizophrenia (Zhang et al., 2010).

Transcriptome-wide association studies (TWAS) present a means of addressing gene-disease associations by integrating GWAS summary statistics with expression quantitative trait loci (eQTL) data to score a gene based on an association between its predicted expression and a trait (Gusev et al., 2016). A recent TWAS study identified the expression of a long noncoding RNA (lncRNA) transcribed antisense to *HTR5A, HTR5A-AS1*, to be significantly associated with schizophrenia risk (Collado-Torres et al., 2019). Collado-Torres et al. profiled 900 postmortem brain tissue samples from the hippocampus (*n* = 447) and dorsolateral prefrontal cortex (dlPFC; *n* = 453) from 286 individuals diagnosed with schizophrenia and 265 not diagnosed with schizophrenia. By integrating the most recent schizophrenia GWAS and their own eQTL results, the authors performed brain region-specific TWAS (Collado-Torres et al., 2019) and reported over 1,140 significant associations spanning 333 genes, including two independent associations with *HTR5A-AS1*: a junction-level hippocampal signal (*Z* = −3.99, *p* = 6.52 × 10^−5^, false discovery rate [FDR] = 2.93 × 10^−3^) and an exon-level signal in the dlPFC (*Z* = −3.54, *p* = 4.06 × 10^−4^, FDR = 1.04 × 10^−2^). The significant association of *HTR5A-AS1* in the hippocampus is linked to schizophrenia GWAS variants that are predicted to act as eQTLs regulating its expression. Nevertheless, *HTR5A-AS1* remains largely unexplored in the literature, with PubMed (National Center for Biotechnology Information, 2025) and GeneCards (GeneCards, 2025) searches showing no PubMed-indexed publications explicitly mentioning it to date. In this study, the dlPFC was prioritized for postmortem analyses because it was one of the two regions in which *HTR5A-AS1* showed significant TWAS associations in Collado-Torres et al. (2019). Furthermore, the dlPFC is one of the most consistently implicated cortical regions in schizophrenia, particularly in relation to executive dysfunction and working-memory impairment (Karlsgodt et al., 2010). Its large and well-powered BrainSeq sample size further makes it an optimal region for detecting case–control transcriptional differences.

## 2 Materials and methods

### 2.1 Transcriptome-wide association study

R (R Core Team, 2025b) version 4.4.3 with the readr (CRAN, 2025e), dplyr (CRAN, 2025b), ggplot2 (Wickham, 2016), and stringr (CRAN, 2025g) packages was used to analyze Collado-Torres et al. TWAS results, which were downloaded from http://eqtl.brainseq.org/phase2/. The feature-level TWAS results were imported, and gene identifiers were cleaned by removing Ensembl version suffixes to allow for consistent matching across datasets. The data were then filtered to include only rows containing *HTR5A-AS1* or its Ensembl ID: ENSG00000220575. TWAS *p*-values were converted to a numeric format, and the minimum *p*-value across brain regions was identified and reported to summarize the strongest transcriptome-wide association for *HTR5A-AS1*. This step was performed to establish whether *HTR5A-AS1* showed robust statistical evidence for involvement in schizophrenia across the transcriptome.

Parallel to the analysis of *HTR5A-AS1*, the same procedure was applied to the paired sense gene *HTR5A*. TWAS summary statistics were imported and filtered using both the gene symbol and Ensembl identifier (ENSG00000157219), and the corresponding *Z*-scores and *p*-values for hippocampus and dlPFC were extracted. This step was performed to determine whether *HTR5A* itself shows a transcriptome-wide association with schizophrenia. As with *HTR5A-AS1*, all *p*-values were converted to numeric format, and brain region–specific results were evaluated. These findings are reported in the Results section for comparison with the antisense RNA.

### 2.2 Linkage disequilibrium analysis

LD statistics were obtained using the LDlinkR (Myers et al., 2020) package in R, which provides programmatic access to the NIH LDlink suite (Lin et al., 2021). The LDpair() function was used to calculate pairwise LD between the schizophrenia GWAS sentinel SNP (rs1583830) and the lead exon-level eQTL SNP (rs1881691). Analyses were restricted to the European (EUR) reference population from the 1000 Genomes Project Phase 3 under the GRCh38 genome build. LD metrics reported include *D*′, *r*^2^, and *p*-values. This analysis was performed to evaluate whether the schizophrenia risk SNP and the eQTL regulating *HTR5A-AS1* are linked on the same haplotype, suggesting a shared genetic mechanism.

### 2.3 Postmortem expression analysis (BrainSeq)

Bulk RNA-seq expression data containing raw count data and sample metadata from postmortem brain tissue were loaded into R. The SummarizedExperiment (Bioconductor, 2025), dplyr (CRAN, 2025b), ggplot2 (Wickham, 2016), forcats (CRAN, 2025c), and ggbeeswarm (CRAN, 2025d) packages were used to extract expression values for *HTR5A-AS1* and its sense partner *HTR5A*, which were converted to counts per million (CPM) using sample-specific library sizes. The samples were filtered to include only adult donors (at least 18 years old) from the hippocampus and dlPFC. No additional covariates were included; comparisons were restricted by age and brain region and conducted using non-parametric testing. Expression values were log_2_-transformed as log_2_(CPM+1) prior to statistical testing, and group differences between control and schizophrenia samples were assessed using Wilcoxon rank-sum tests. To address potential sex effects, all analyses were repeated after stratifying by biological sex (male vs. female), using the same pipeline, and Wilcoxon rank-sum tests were performed within each sex group for both *HTR5A-AS1* and its sense gene *HTR5A*. Sex-stratified adult subsets were generated for hippocampus and dlPFC, and violin plots were produced to visualize these comparisons. These sex-stratified results are presented in Supplementary Figures S1–S4. This analysis directly tested whether *HTR5A-AS1* and *HTR5A* show region- and sex-specific dysregulation in disease-relevant cortical and limbic regions.

### 2.4 Developmental expression trajectories (BrainSpan)

Expression trajectories of *HTR5A-AS1* were derived from BrainSpan bulk RNA-seq data. Transcript abundance values were extracted from the expression matrix using gene annotations from the accompanying metadata. Reported sample ages were parsed into post-conception days (PCD) by converting gestational weeks, months, and years into a continuous day scale, with birth defined at 280 days. Expression values were plotted against log_10_-scaled PCD, with vertical reference lines marking birth and selected developmental timepoints.

A linear regression model (TPM ~ PCD) was fit to quantify the global developmental trend. The regression slope (TPM/day), coefficient of determination (*R*^2^), and *p*-value were calculated using the broom (CRAN, 2025a) package and annotated directly onto the plot. The slope serves as a quantitative measure of whether *HTR5A-AS1* expression systematically increased or decreased across human brain development.

Sample metadata were mapped from BrainSpan structure acronyms into broad anatomical classes: NCX, hippocampus, STR, MD, AMY, and CBC. Regions with the six highest median TPM values were retained for visualization. For each of the six regions, linear regression models (TPM ~ PCD) were fit independently. Slopes (TPM change per post-conception day) were extracted using the broom package, and slope values were embedded into the figure legend for interpretability. Points were overlaid on regression lines, with a log-scaled *x*-axis and vertical markers at birth and selected guide marks, enabling identification of the most developmentally dynamic brain regions for *HTR5A-AS1* expression. Because BrainSeq and BrainSpan use different normalization frameworks (log_2_(CPM + 1) vs. TPM), all analyses were conducted within each dataset, and absolute expression values were not directly compared across resources.

### 2.5 UCSC genome browser validation

The UCSC Genome Browser (hg38 assembly, accessed 2025) (UCSC Genome Browser, 2025) was used to confirm the presence and transcriptional structure of *HTR5A-AS1*. The locus was examined with the following tracks enabled: Base Position, database of single nucleotide polymorphisms (dbSNP) build 155 (to visualize genome-wide significant GWAS SNP rs1583830 and the lead eQTL SNP rs1881691), GENCODE v48 annotation, CLS transcript models, Adult Blood/Brain transcript models, Cell Line and Embryonic Brain transcript models, ENCODE4 long-read transcripts, and GTEx tissue expression. A PDF export of the genome browser view was generated to document transcript models and SNP placement relative to *HTR5A-AS1*.

### 2.6 Functional predictions (lncHUB)

Functional predictions for *HTR5A-AS1* were obtained from the lncHUB platform (lncHUB, 2025), including Kyoto Encyclopedia of Genes and Genomics (KEGG) pathway *Z*-scores, Mammalian/MGI mouse phenotype *Z*-scores, Gene Ontology (GO) biological process *Z*-scores, and co-expressed gene *Z*-scores. The exported result tables were read into R and processed in dplyr; display names were wrapped for readability and converted to title case while preserving acronyms (e.g., GPCR, GABA). For each category, the top entries by *Z*-score were selected without transformation (KEGG top 15; Mouse Phenotype top 15; GO Biological Process top 20; Co-expression top 25). Ranked horizontal bar charts were generated in ggplot2, with bars representing the reported *Z*-scores and numeric labels printed just beyond bar ends. Axis text was brought closer to bars to improve legibility, and figure margins were adjusted to avoid clipping of labels. No statistical re-analysis or rescaling of *Z*-scores was performed; ordering and formatting were applied for visualization. All plots were produced in R [packages: tibble (CRAN, 2025h), dplyr, ggplot2, stringr, forcats, rlang (CRAN, 2025f), grid (R Core Team, 2025a)] using fixed fill colors and identical geometries across panels to facilitate comparison.

## 3 Results

### 3.1 TWAS identifies *HTR5A-AS1* association with schizophrenia

**Figure 1:**
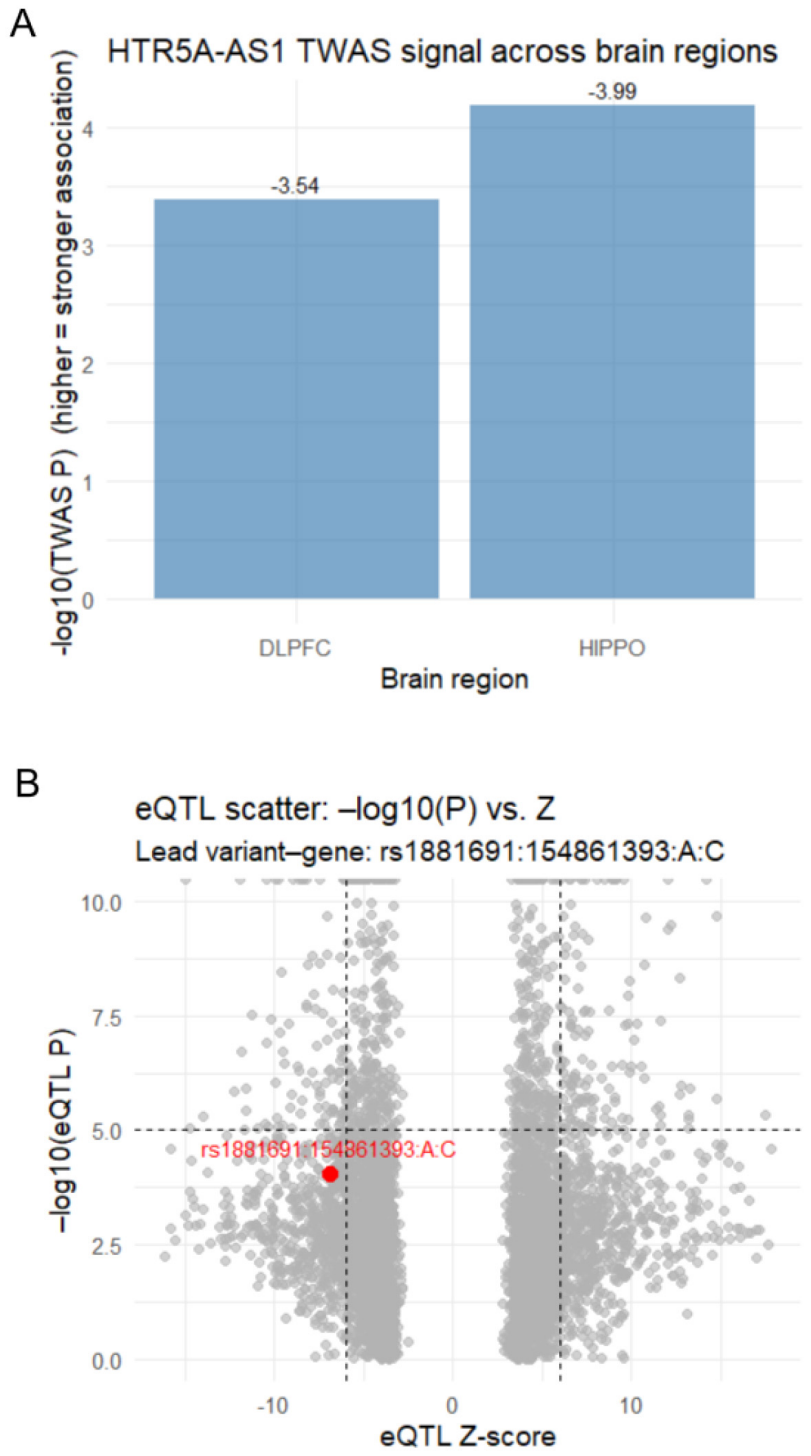
*HTR5A-AS1* TWAS results and eQTL associations with schizophrenia. **(A)** Bar plot showing −log_10_(p-value) for *HTR5A-AS1* in dorsolateral prefrontal cortex (dlPFC) and hippocampus (HIPPO) from transcriptome-wide association studies (TWAS) analysis (Collado-Torres et al., 2019). *HTR5A-AS1* showed its strongest association in the HIPPO (*p* = 6.52 × 10^−5^, FDR-adjusted *p* = 2.93 × 10^−3^), exceeding the false discovery rate (FDR) threshold for significance, and a weaker but notable association in dlPFC (*p* = 4.06 × 10^−4^, FDR-adjusted *p* = 1.04 × 10^−2^). **(B)** Expression quantitative trait loci (eQTL) association for *HTR5A-AS1* with lead SNP rs1881691. Scatterplot showing eQTL *Z*-scores (x-axis) versus −log_10_(p-values) (y-axis) across all tested variants. The lead variant rs1881691 (highlighted in red) shows a strong association with *HTR5A-AS1* expression (*Z* = −6.92, *p* = 9.15 × 10^−5^) in the HIPPO samples.

The first step of this study was to determine whether *HTR5A-AS1* is associated with schizophrenia on a genetic and expression level, as establishing such links is critical before pursuing functional interpretation. In the hippocampus, the strongest TWAS association with *HTR5A-AS1* was detected at the junction level (*Z* = −3.99, *p* = 6.52 × 10^−5^, FDR = 2.93 × 10^−3^; Figure 1A) (Collado-Torres et al., 2019). Junction-level hits indicate that genetic regulation influences exon–exon splicing events, implicating *HTR5A-AS1* isoform usage in disease susceptibility. The lead GWAS variant in this locus, rs1583830, showed robust association with schizophrenia (GWAS *Z* = 4.73, *p* = 2.25 × 10^−6^). The corresponding hippocampal eQTL, rs1881691, exerted a strong effect on *HTR5A-AS1* expression (eQTL *Z* = −5.71, *p* = 9.15 × 10^−5^; Figure 1B). In the dlPFC, an exon-level feature of *HTR5A-AS1* also reached statistical significance (TWAS *Z* = −3.54, *p* = 4.06 × 10^−4^, FDR = 1.04 × 10^−2^; Figure 1A). Exon-level hits capture expression changes, reflecting the regulatory effects of transcript expression level rather than isoform usage. The same GWAS sentinel single-nucleotide polymorphism (SNP), rs1583830, again showed strong association with schizophrenia, while the lead exon eQTL, rs1881691, exhibited an even stronger effect in this region (eQTL *Z* = −6.92, *p* = 9.15 × 10^−5^; Figure 1B). Across both regions, the GWAS sentinel SNP (rs1583830) and the exon-level eQTL SNP (rs1881691) were found to be in moderate-to-strong linkage disequilibrium (LD; *D*′ = 0.82, *r*^2^ = 0.60, *p* = 1.0 × 10^−4^), suggesting that the same haplotype block drives the schizophrenia risk association and *HTR5A-AS1* regulation. Taken together, these results indicate that the haplotype containing rs1583830 and rs1881691 regulates *HTR5A-AS1* expression and splicing, supporting its role as a schizophrenia risk gene. For comparison, parallel TWAS analysis of the paired sense gene *HTR5A* revealed no transcriptome-wide significant associations in either region. In the hippocampus, the strongest *HTR5A* feature showed a *Z*-score of –2.44 and nominal *p* = 0.015 (FDR = 0.113), while in the dlPFC the corresponding feature showed a *Z*-score of –1.27 and *p* = 0.204 (FDR = 0.502). All values fell below false-discovery thresholds, confirming that the TWAS signal in this locus is specific to the antisense transcript *HTR5A-AS1*.

### 3.2 Reduced *HTR5A-AS1* expression in the hippocampus

**Figure 2:**
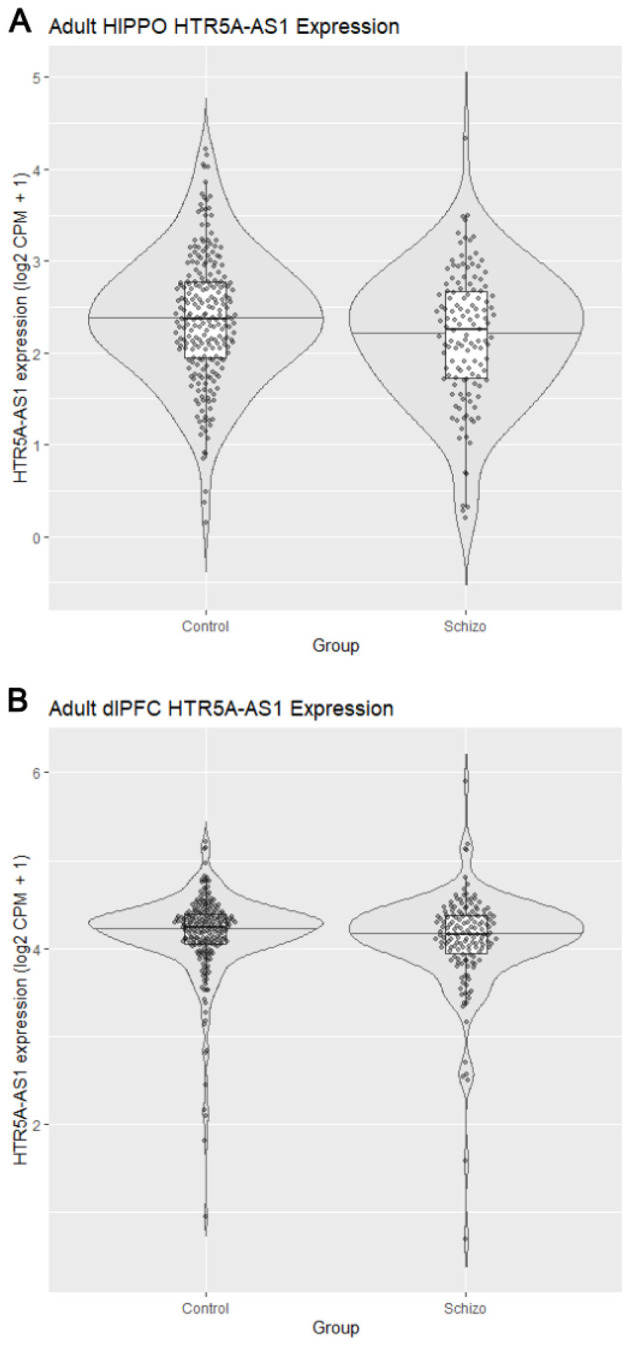
Violin plots of regional *HTR5A-AS1* expression using Collado-Torres et al. (2019) TWAS data (Collado-Torres et al., 2019). **(A)** Violin plot showing log_2_(counts per million [CPM]+1) *HTR5A-AS1* expression in adult hippocampus (HIPPO) from postmortem schizophrenia and control donors. Data were normalized to CPM using sample-specific library sizes. A Wilcoxon rank-sum test revealed significantly lower expression in schizophrenia (*p* = 0.0193). **(B)** Violin plot showing log_2_(CPM+1) *HTR5A-AS1* expression in adult dorsolateral prefrontal cortex (dlPFC) from postmortem schizophrenia and control donors (*p* = 0.0954, Wilcoxon rank-sum test). Corresponding sex-stratified analyses for both regions are provided in Supplementary Figures S1, S2.

Analyses of postmortem raw count RNA sequencing (RNA-seq) data from 900 brain samples across hippocampus (*n* = 447) and dlPFC (*n* = 453), from both control and schizophrenia donors, were performed (Collado-Torres et al., 2019). In the hippocampus, *HTR5A-AS1* was expressed significantly lower (*p* = 0.0193, Wilcoxon rank-sum test; Figure 2A) in schizophrenia than in the control samples, consistent with its negative TWAS *Z*-score, −3.99. In the dlPFC, expression was lower in schizophrenia but did not reach statistical significance (*p* = 0.0954, Wilcoxon rank-sum test; Figure 2B). These findings indicate that *HTR5A-AS1* is significantly downregulated in the hippocampus of schizophrenia donors, consistent with genetic predictions of disease risk, and motivated follow-up sex-stratified analyses (Supplementary Figures S1, S2).

### 3.3 Sex-stratified postmortem expression of *HTR5A-AS1* and *HTR5A*

Given prior evidence for sex differences in serotonergic signaling (Groves et al., 2013), all postmortem analyses were repeated after stratifying by biological sex. In the hippocampus, *HTR5A-AS1* showed nominally lower expression in male patients compared with male controls (*p* = 0.0731; Supplementary Figure S1), whereas no significant difference was observed in females (*p* = 0.1785; Supplementary Figure S1). In the dlPFC, *HTR5A-AS1* showed no significant case-control differences in either female (*p* = 0.7322; Supplementary Figure S2) or male (*p* = 0.0871; Supplementary Figure S2) samples.

Although *HTR5A* itself did not emerge as a significant TWAS hit, whether or not the receptor displayed similar expression patterns in postmortem tissue was tested. In the hippocampus, *HTR5A* expression was modestly but significantly reduced in schizophrenia when all adults were considered together (*p* = 0.0182), with this effect driven primarily by males (*p* = 0.0406; Supplementary Figure S3) and not significant in females (*p* = 0.2548; Supplementary Figure S3). By contrast, *HTR5A* expression in the dlPFC was not significantly different between schizophrenia and control donors in the combined (*p* = 0.6581), female-only (*p* = 0.7752; Supplementary Figure S4), or male-only (*p* = 0.6589; Supplementary Figure S4) samples. Together, these results suggest a region- and sex-specific relationship in which hippocampal downregulation of both *HTR5A-AS1* and *HTR5A* is strongest in male schizophrenia donors.

### 3.4 Region-specific expression in the human brain

**Figure 3:**
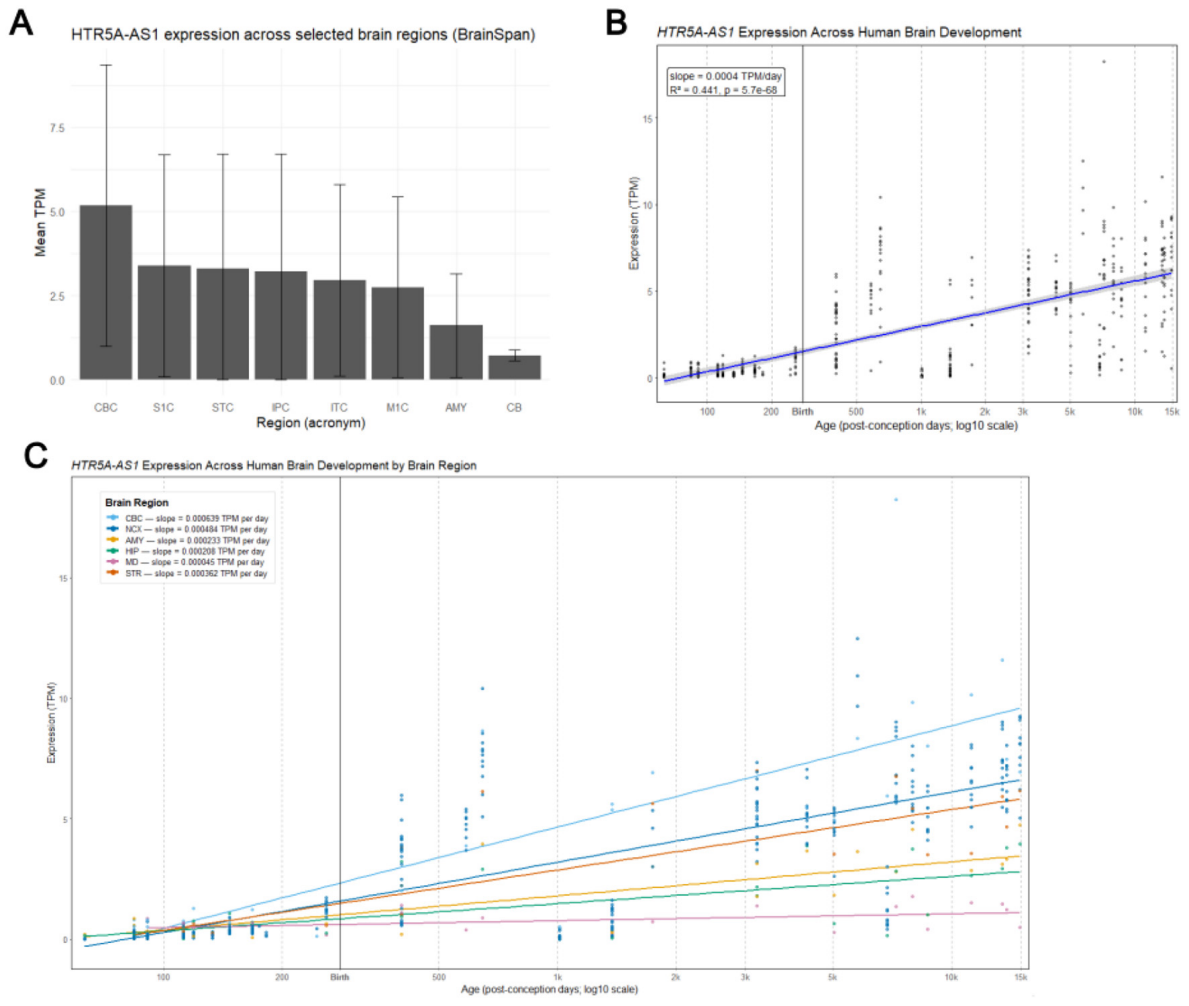
BrainSpan RNA-seq *HTR5A-AS1* expression by brain region, across development (BrainSpan Atlas of the Developing Human Brain, 2025). **(A)** Mean TPM values are shown for selected brain regions, ordered by decreasing expression. **(B)** Expression of *HTR5A-AS1* increases steadily from prenatal to postnatal stages in BrainSpan RNA-seq data. A linear regression shows a significant positive trajectory (slope = 0.0004 TPM/day; *R*^2^ = 0.44; *p* = 5.7 × 10^−68^), indicating strong developmental regulation. **(C)** Region-stratified regression shows distinct developmental patterns.

To evaluate whether *HTR5A-AS1* is expressed in regions most consistently disrupted in schizophrenia, its expression patterns across brain regions were analyzed using RNA-seq data from BrainSpan Atlas of the Developing Human Brain (2025). Expression was quantified as transcripts-per-million (TPM). Expression was highest in the cerebellar cortex (CBC; mean = 5.17 TPM, median = 5.03, *n* = 29), followed by the primary somatosensory cortex (S1C; mean = 3.38 TPM, median = 2.45, *n* = 26), the posterior superior temporal cortex (STC; mean = 3.30 TPM, median = 1.52, *n* = 36), and the inferior parietal cortex (IPC; mean = 3.22 TPM, median = 1.24, *n* = 33). Other notable regions with moderate expression included the inferolateral temporal cortex (ITC; mean = 2.95 TPM, *n* = 34) and the primary motor cortex (M1C; mean = 2.75 TPM, *n* = 26), whereas expression was comparatively lower in the amygdaloid complex (AMY; mean = 1.62 TPM, *n* = 33) and especially the cerebellum (CB; mean = 0.73 TPM, *n* = 3) (Figure 3A). This analysis confirmed that *HTR5A-AS1* shows strong region-specific enrichment, particularly in cortical and cerebellar regions.

### 3.5 Developmental trajectory of *HTR5A-AS1*

To determine whether *HTR5A-AS1* expression is developmentally regulated, brain RNA-seq data spanning prenatal to adult stages were analyzed from BrainSpan (BrainSpan Atlas of the Developing Human Brain, 2025). Developmental analyses showed dynamic changes across the lifespan. Linear regression across all samples revealed an overall positive developmental trajectory (β≈0.0004 TPM/day; Figure 3B). Region-stratified linear fits showed that *HTR5A-AS1* expression rises at distinct rates across brain regions (Figure 3C). The fastest rate of increase was observed in the CBC (β = 0.000639 TPM/day), followed by the neocortex (NCX; β = 0.000484) and striatum (STR; β = 0.000362). Moderate increases were seen in the AMY (β = 0.000233) and hippocampus (β = 0.000208), while the mediodorsal nucleus of the thalamus (MD) showed the lowest rates (β = 0.000045), suggesting relative developmental stability.

### 3.6 Transcript validation via UCSC Genome Browser and co-expression analysis

**Figure 4:**
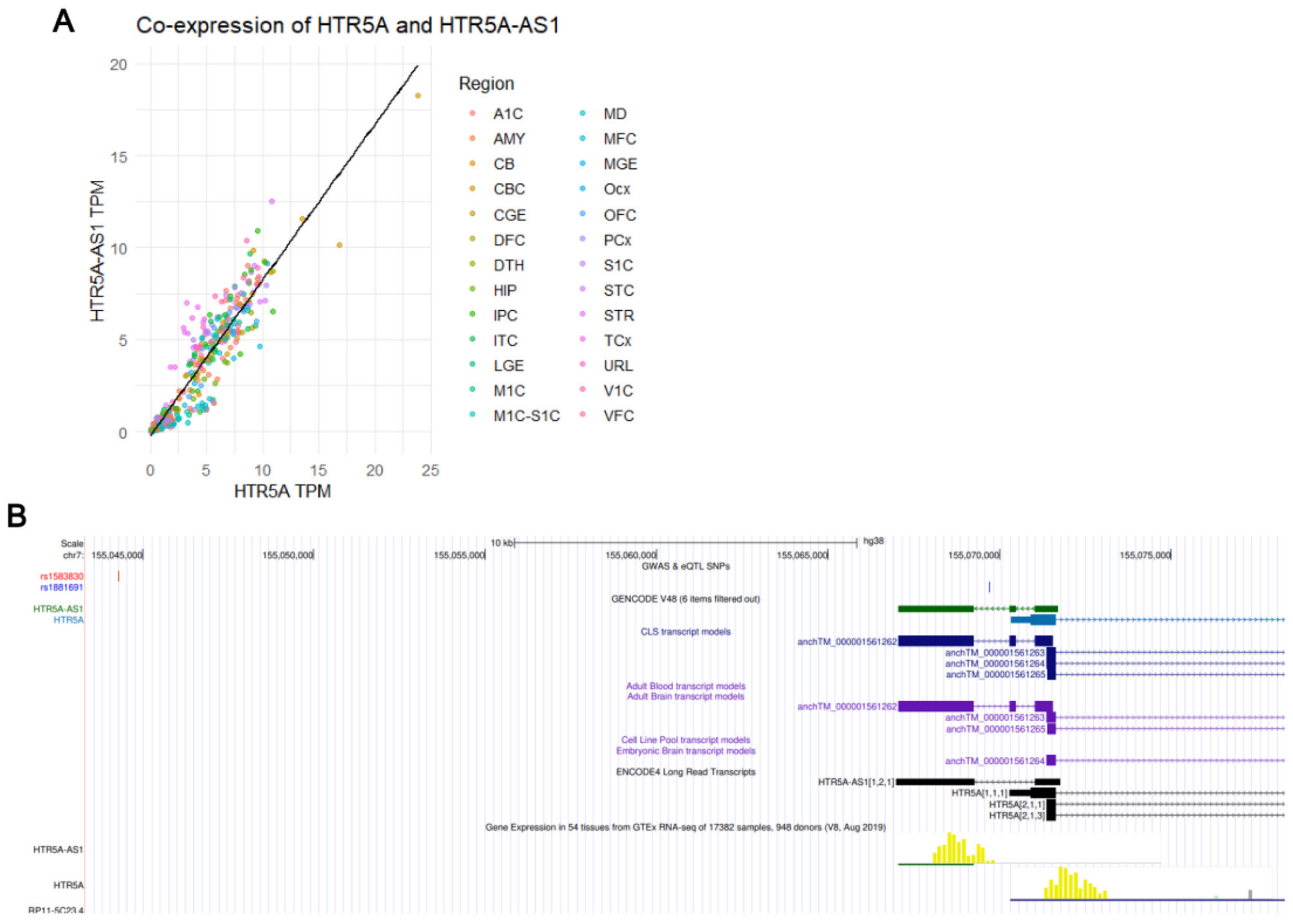
Co-expression with *HTR5A* and UCSC Genome Browser analysis. **(A)** Scatterplot showing TPM for *HTR5A-AS1* (y-axis) vs. *HTR5A* (x-axis) across samples in the BrainSpan dataset (BrainSpan Atlas of the Developing Human Brain, 2025), colored by brain region. Pearson's *r* = 0.943, *p* < 2.2 × 10^−16^. **(B)** UCSC Genome Browser (hg38) snapshot of the *HTR5A-AS1* locus on chromosome 7 (UCSC Genome Browser, 2025). Displayed tracks include genome-wide association studies (GWAS) and eQTL sentinel SNPs (rs1583830, red; rs1881691, blue), GENCODE v48 annotation, transcript models, ENCODE4 long-read RNA-seq, and GTEx RNA-seq.

To verify that *HTR5A-AS1* is a genuine transcript rather than an artifact of transcriptional noise, its annotation and expression were examined using independent datasets. In the UCSC Genome Browser analysis (hg38 assembly; accessed 2025) (UCSC Genome Browser, 2025), long-read RNA-seq tracks confirmed multi-exonic transcripts in brain tissues overlapping the GENCODE-annotated locus. The schizophrenia-associated SNPs appeared upstream of the *HTR5A-AS1* locus, consistent with potential regulatory positioning. These data support *HTR5A-AS1* as a distinct multi-exonic antisense transcript to *HTR5A* (Figure 4B). To test whether *HTR5A-AS1* expression is related to its sense strand partner, *HTR5A*, the same BrainSpan dataset was examined (BrainSpan Atlas of the Developing Human Brain, 2025). Across 524 samples, *HTR5A-AS1* expression correlated strongly with *HTR5A* (Pearson's *r* = 0.943, *p* < 2.2 × 10^−16^; Figure 4A).

### 3.7 Predicted functional associations of *HTR5A-AS1*

Pathway enrichment and co-expression analyses from lncHUB (lncHUB, 2025) indicated a potentially strong enrichment for pathways related to synaptic transmission, learning, and addiction (Supplementary Figure S5). The top KEGG pathway was amyotrophic lateral sclerosis (ALS; *Z* = 6.354), followed by nicotine addiction (*Z* = 5.917) and long-term potentiation (*Z* = 5.653). Predicted mouse phenotypes highlighted associations with abnormal short-term spatial reference memory (*Z* = 7.54), abnormal hearing electrophysiology (*Z* = 7.20), and abnormal peripheral synaptic transmission (*Z* = 6.93; Supplementary Figure S6). Gene Ontology (GO) enrichment identified GABAergic synaptic transmission (*Z* = 7.943), regulation of secretion by cell (*Z* = 6.449), and gamma-aminobutyric acid signaling pathway (*Z* = 6.396) among the top biological processes (Supplementary Figure S7). Analysis of the top 500 co-expressed genes revealed many known synaptic and neuronal regulators, including *HTR5A* (*Z* = 0.948), *CAMK2A* (*Z* = 0.896), *SNAP25* (*Z* = 0.880), and *GRIN1* (*Z* = 0.871; Supplementary Figure S8). These enrichment patterns should be interpreted cautiously, as they derive from co-expression and statistical associations rather than experimental evidence. These analyses alone do not prove that *HTR5A-AS1* directly influences GABAergic or synaptic pathways. Full lncHUB bar plots are provided in Supplementary Figures S5–S8.

## 4 Discussion

This study provides the first detailed characterization of a lncRNA transcribed antisense to the serotonin receptor gene *HTR5A, HTR5A-AS1*, in the context of schizophrenia. TWAS analyses (Collado-Torres et al., 2019) identified two significant associations between *HTR5A-AS1* expression and schizophrenia: a junction-level signal in the hippocampus and an exon-level signal in the dlPFC. Beyond genetic associations, expression analyses revealed significantly reduced *HTR5A-AS1* expression in the hippocampus of schizophrenia brain donors, aligning with TWAS predictions (Collado-Torres et al., 2019). While the reduction in the dlPFC did not reach statistical significance, the trend aligns with the negative TWAS association. Notably, the TWAS associations were identified at the junction and exon level, suggesting that schizophrenia risk may involve altered isoform usage of *HTR5A-AS1*. Such splicing-dependent effects would be diluted in bulk gene-level differential expression analyses, underscoring the need for isoform-aware approaches.

Using BrainSpan data (BrainSpan Atlas of the Developing Human Brain, 2025), analyses revealed that *HTR5A-AS1* is developmentally regulated, with distinct region-specific trajectories. Consistent with schizophrenia's developmental origins, *HTR5A-AS1* displayed steep increases in expression in the cerebellar cortex (CBC) and neocortex (NCX), moderate increases in the hippocampus and AMY, and relative stability in the thalamus. These trajectories suggest that *HTR5A-AS1* may contribute to the maturation of cortical–cerebellar and cortico-limbic circuits, which are strongly implicated in schizophrenia's cognitive and affective dysfunction. The differing developmental slopes observed in the AMY and MD also carry functional implications. The AMY, which showed a moderate rate of increase, is central to affective regulation, emotional salience processing, and fear learning—domains frequently altered in schizophrenia. By contrast, the MD exhibited relative developmental stability, consistent with its role as a relay hub with less protracted maturation. These region-specific patterns suggest that *HTR5A-AS1* may participate in shaping both affective (amygdala-driven) and cognitive-attentional (thalamic–cortical) circuits relevant to schizophrenia.

The possibility that *HTR5A-AS1* is simply transcriptional noise arising from *HTR5A* was also refuted. This study provides multiple independent lines of evidence to validate the transcript's authenticity: multi-exonic structure validated by long-read RNA-seq, consistent annotation in GENCODE, and strong independent expression signals across multiple datasets (UCSC Genome Browser, 2025). Its co-expression with *HTR5A* is expected for antisense pairs but does not negate potential regulatory function. Instead, it raises the possibility of cis-regulation of *HTR5A*, as well as coordinated involvement in common pathways.

Functional predictions and co-expression analyses consistently implicated synaptic and cognitive processes, notably GABAergic signaling and long-term potentiation (lncHUB, 2025). These findings align with the developmental regulation and cortical–hippocampal enrichment of *HTR5A-AS1*, regions in which disruptions of inhibitory–excitatory balance are strongly implicated in schizophrenia. The lncHUB predictions differentiated between “GABAergic synaptic transmission” and the broader “GABAergic signaling pathway,” which carry distinct biological meanings. GABAergic signaling encompasses receptor-mediated intracellular cascades and downstream regulatory pathways, whereas GABAergic synaptic transmission refers to presynaptic vesicle release, reuptake, and inhibitory synaptic homeostasis. Schizophrenia is known to involve disruptions at both levels, including impaired inhibitory interneuron function and altered GABA receptor dynamics. The enrichment of *HTR5A-AS1* in both categories suggests that it may influence multiple components of inhibitory circuitry, although the present findings remain correlative.

Together, these results are consistent with the possibility that *HTR5A-AS1* may modulate neuronal excitability or plasticity in a region-specific manner, potentially by regulating *HTR5A* expression in the hippocampus or by interacting with other synaptic genes such as *CAMK2A, SNAP25*, and *GRIN1*. However, the present study did not include a dedicated differential expression analysis testing directional consistency between lncHUB-identified synaptic plasticity genes and *HTR5A-AS1*. A systematic assessment of these synaptic plasticity gene sets therefore falls outside the scope of the current work but represents an important direction for future studies aiming to test (through e.g., gene-set-level differential expression modeling) whether the observed enrichment patterns correspond to convergent transcriptional alterations in schizophrenia.

Sex-stratified postmortem analyses further refine this picture. In the hippocampus, significant downregulation of both *HTR5A-AS1* and *HTR5A* was present in male schizophrenia donors, whereas effects were weaker and non-significant in females. No robust expression differences were observed for either transcript in the dlPFC when stratified by sex. These findings are consistent with reports of sex-dependent serotonergic alterations in psychiatric disease (Groves et al., 2013) and suggest that *HTR5A-AS1* may contribute to schizophrenia risk in a regionally and sexually dimorphic manner.

Importantly, although *HTR5A-AS1* showed robust TWAS associations, the sense gene *HTR5A* did not reach transcriptome-wide significance in the hippocampus or dlPFC. This finding is fully consistent with prior studies showing that *HTR5A* dysregulation in schizophrenia is often observed at the level of receptor availability or signaling rather than bulk mRNA abundance. As a GPCR, *HTR5A* is strongly shaped by post-transcriptional and translational regulation, including alternative splicing, RNA editing, and potential antisense-mediated repression. Therefore, the absence of a TWAS signal for *HTR5A*, combined with its sex-dependent decrease in hippocampal expression, supports the interpretation that *HTR5A-AS1* may influence schizophrenia risk partly through post-transcriptional modulation of *HTR5A*, not through steady-state transcript changes. This resolves apparent discrepancies with prior reports and strengthens the biological link between the antisense RNA and its paired receptor.

There are several implications and limitations to acknowledge. First, the predicted associations obtained from lncHUB are inherently correlative and based on co-expression (lncHUB, 2025). Although the present study provides convergent evidence supporting *HTR5A-AS1* as a bona fide transcript through integration of genetic association, postmortem expression, developmental trajectory analyses, and co-expression patterns, these findings do not establish a mechanistic role for the lncRNA. All hypothesized functional roles for *HTR5A-AS1* therefore remain putative and cannot be interpreted as causal.

Second, the observation that *HTR5A-AS1* reached transcriptome-wide significance in TWAS while the paired sense-strand receptor gene *HTR5A* did not provides transcript-specific evidence that schizophrenia-associated genetic risk at this locus is preferentially linked to the lncRNA rather than to the receptor transcript itself. However, because the present study did not perform formal colocalization or conditional TWAS analyses (e.g., COLOC, eCAVIAR, RTC), it cannot be concluded whether shared regulatory variants influence multiple transcripts within the locus. Accordingly, the present findings support a primary association with *HTR5A-AS1* while remaining noncommittal about the precise regulatory architecture underlying this signal.

Third, strong co-expression between *HTR5A-AS1* and *HTR5A*, as well as enrichment for synaptic plasticity genes such as *CAMK2A, GRIN1*, and *SNAP25*, complicates functional interpretation. While these genes provide biologically plausible links to synaptic dysfunction in schizophrenia, the present study did not include a dedicated case-control differential expression analysis to determine whether plasticity-related genes show diagnostic effects that are directionally consistent with changes in *HTR5A-AS1*. Evaluating the directionality and convergence of these plasticity gene signatures represents a potentially important direction for future work.

Fourth, all postmortem analyses in this study were performed using bulk-tissue RNA-seq, which averages signals across heterogeneous cell populations. As a result, potential cell-type-specific effects (e.g., alterations restricted to inhibitory interneurons, excitatory projection neurons, and glial populations) may be partially masked or diluted. This limitation is particularly relevant given longstanding evidence for cell-type-specific disruptions of inhibitory-excitatory balance in schizophrenia. Future studies integrating single-cell or spatial transcriptomic approaches will be required to evaluate whether *HTR5A-AS1* exhibits cell-type-specific expression patterns or disease association that cannot be resolved in bulk tissue.

Finally, UCSC Genome Browser inspection and long-read RNA-seq provide strong support for the multi-exonic transcript structure of *HTR5A-AS1* and its genomic context within the schizophrenia-associated locus. While the present study did not aim to systematically resolve the full enhancer–promoter architecture or chromatin-level regulatory mechanisms underlying this region, integrating epigenomic datasets such as ATAC-seq, H3K27ac ChIP-seq, and promoter capture Hi-C will be valuable for refining how schizophrenia-associated variants modulate *HTR5A-AS1* expression.

Taken together, future experimental studies may build on these findings using perturbation approaches—including CRISPR interference, antisense oligonucleotide inhibition, and allele-specific regulatory assays—to test specific regulatory hypotheses involving *HTR5A-AS1*. qPCR validation in independent cohorts and cell-type-specific functional studies may further refine the biological role of this lncRNA. The present study establishes a comprehensive genetic, transcriptional, and developmental framework for *HTR5A-AS1* in schizophrenia, providing a necessary foundation for subsequent mechanistic investigation.

## Data Availability

Public datasets were analyzed in this study (e.g., BrainSpan, GTEx, and BrainSeq/TWAS resources). Source links and accession details are provided in the Methods. Any custom code used for processing and plotting is available from the author upon reasonable request.
