# Supplementary material for: First computational characterization of HTR5A-AS1: a schizophrenia-linked antisense RNA with synaptic functions
Source: Front Neurosci. 2026 Jan 27;20:1716081. doi: 10.3389/fnins.2026.1716081 (PMC12886454; doi:10.3389/fnins.2026.1716081)
Supplement: Supplementary file 1 [file Data_Sheet_1.pdf]

Supplementary Material

SUPPLEMENTARY FIGURES

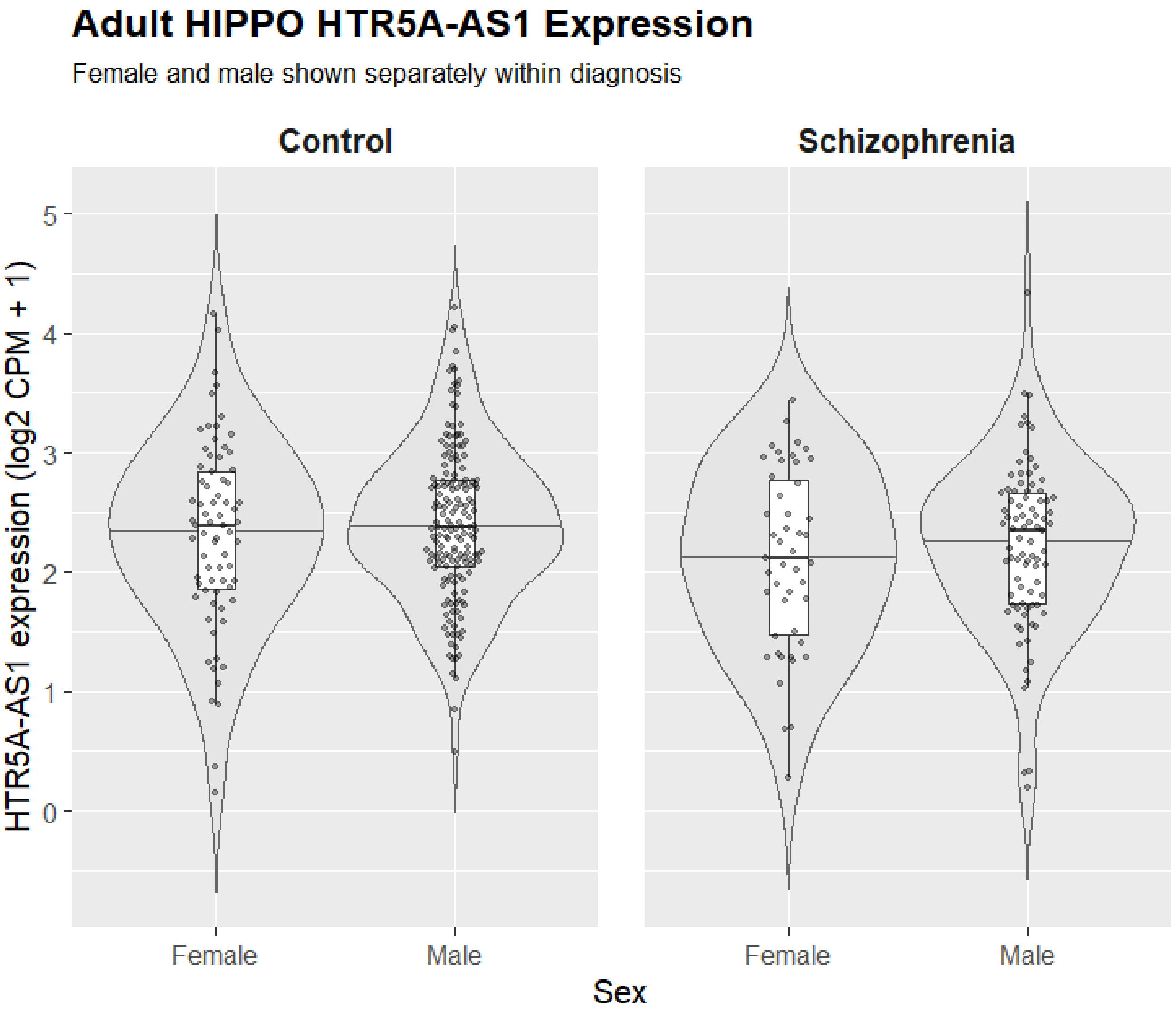

Figure S1. Supplementary Figure S1. Sex-stratified *HTR5A-AS1* expression in adult hippocampus. Violin plots show $\log_2(\mathrm{CPM}+1)$ expression in control and schizophrenia donors, separated by biological sex (female and male). P-values from Wilcoxon rank-sum tests are shown for each sex.

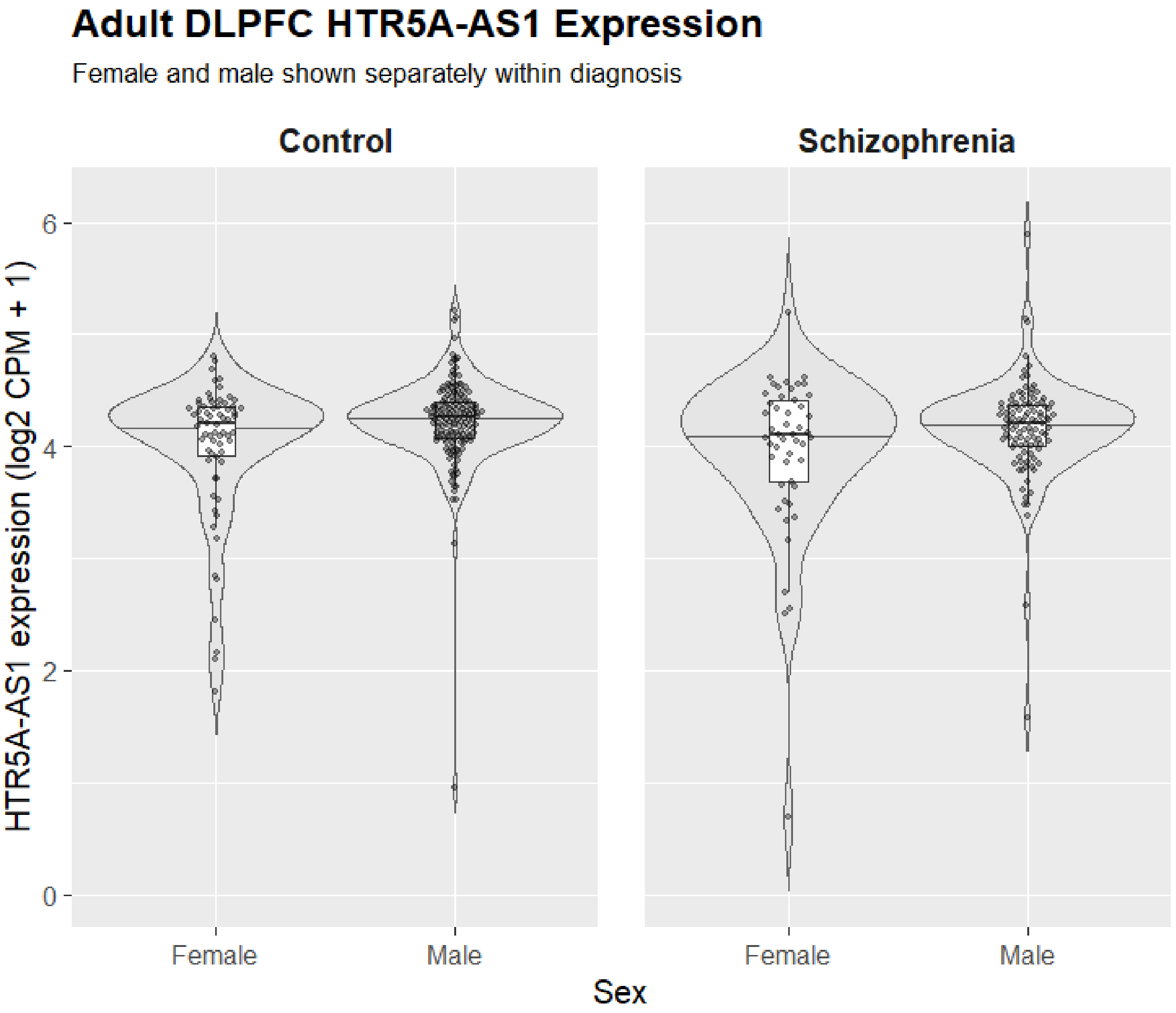

Figure S2. **Supplementary Figure S2.** Sex-stratified *HTR5A-AS1* expression in adult dorsolateral prefrontal cortex (dlPFC). Violin plots show $\log_2(\mathrm{CPM} + 1)$ expression by diagnosis and sex.

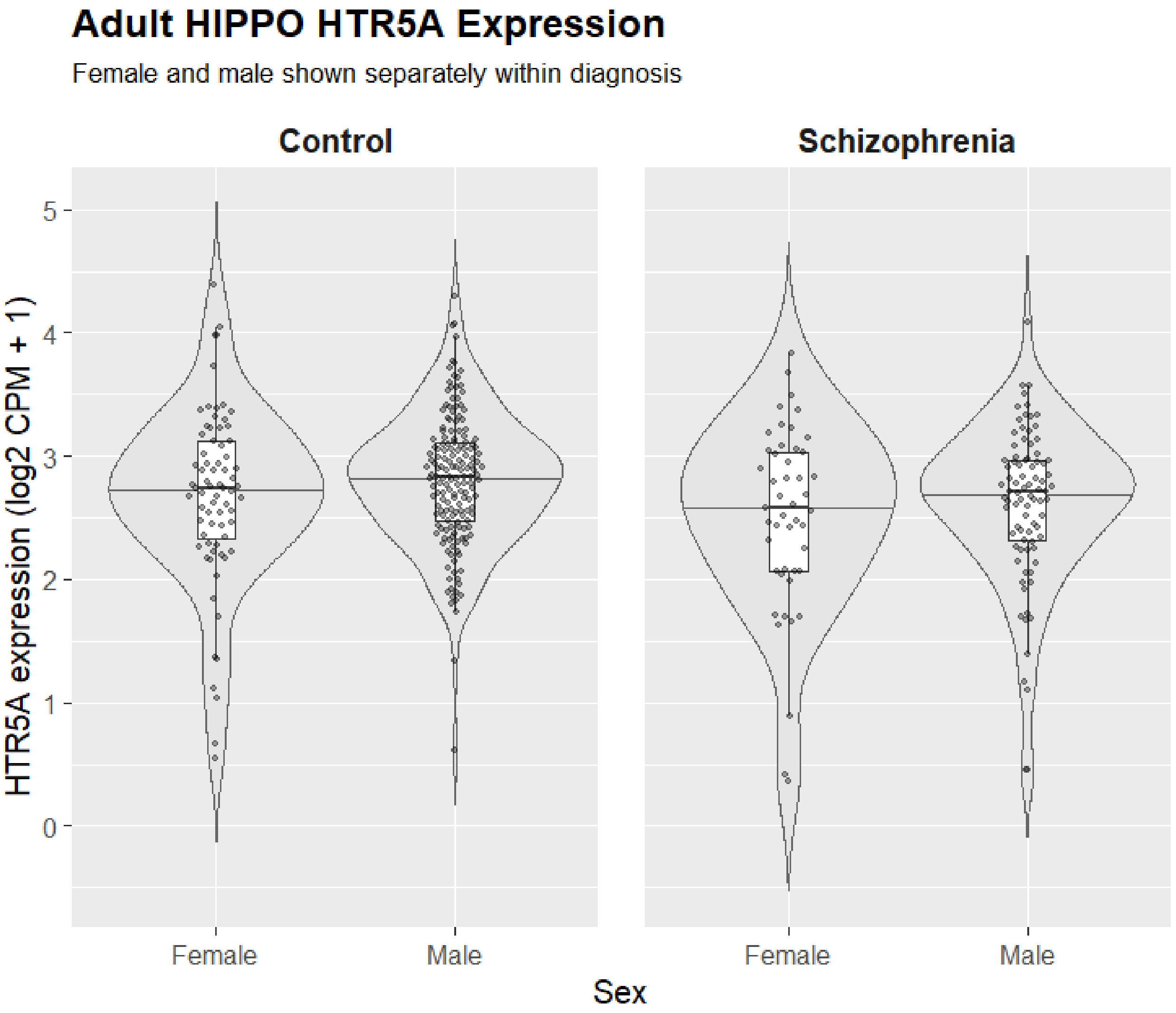

Figure S3. **Supplementary Figure S3.** Sex-stratified *HTR5A* expression in adult hippocampus. Violin plots show $\log_2(\mathrm{CPM} + 1)$ expression in control and schizophrenia donors, with separate distributions for female and male samples.

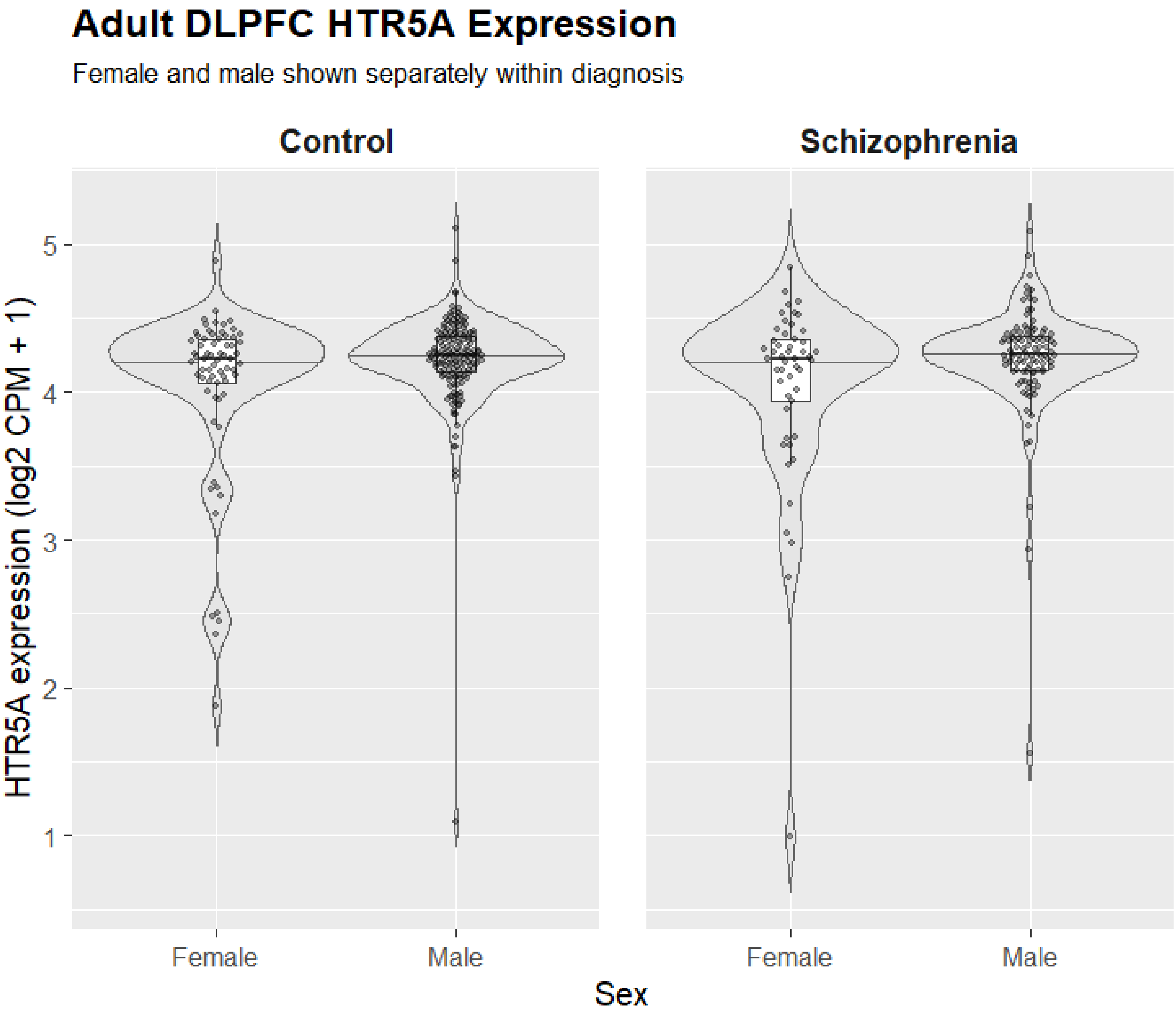

Figure S4. Supplementary Figure S4. Sex-stratified *HTR5A* expression in adult dlPFC. No significant case–control differences were detected in any sex-stratified comparison.

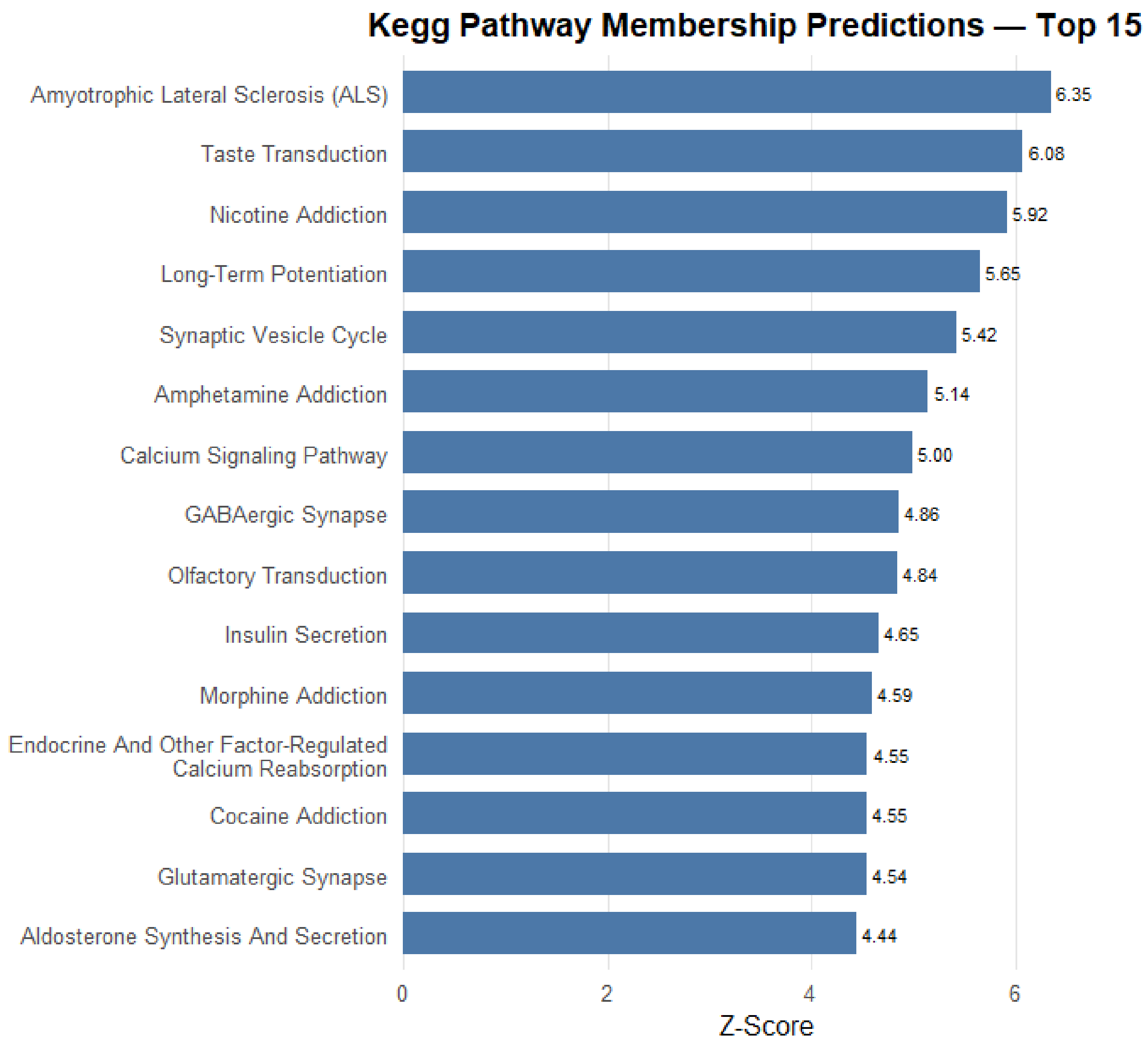

Figure S5. Supplementary Figure S5. lncHUB Kyoto Encyclopedia of Genes and Genomics (KEGG) pathway enrichment for *HTR5A-AS1* based on co-expression. The highest-scoring pathway was amyotrophic lateral sclerosis (ALS; Z = 6.354), followed by nicotine addiction (Z = 5.917) and long-term potentiation (Z = 5.653).

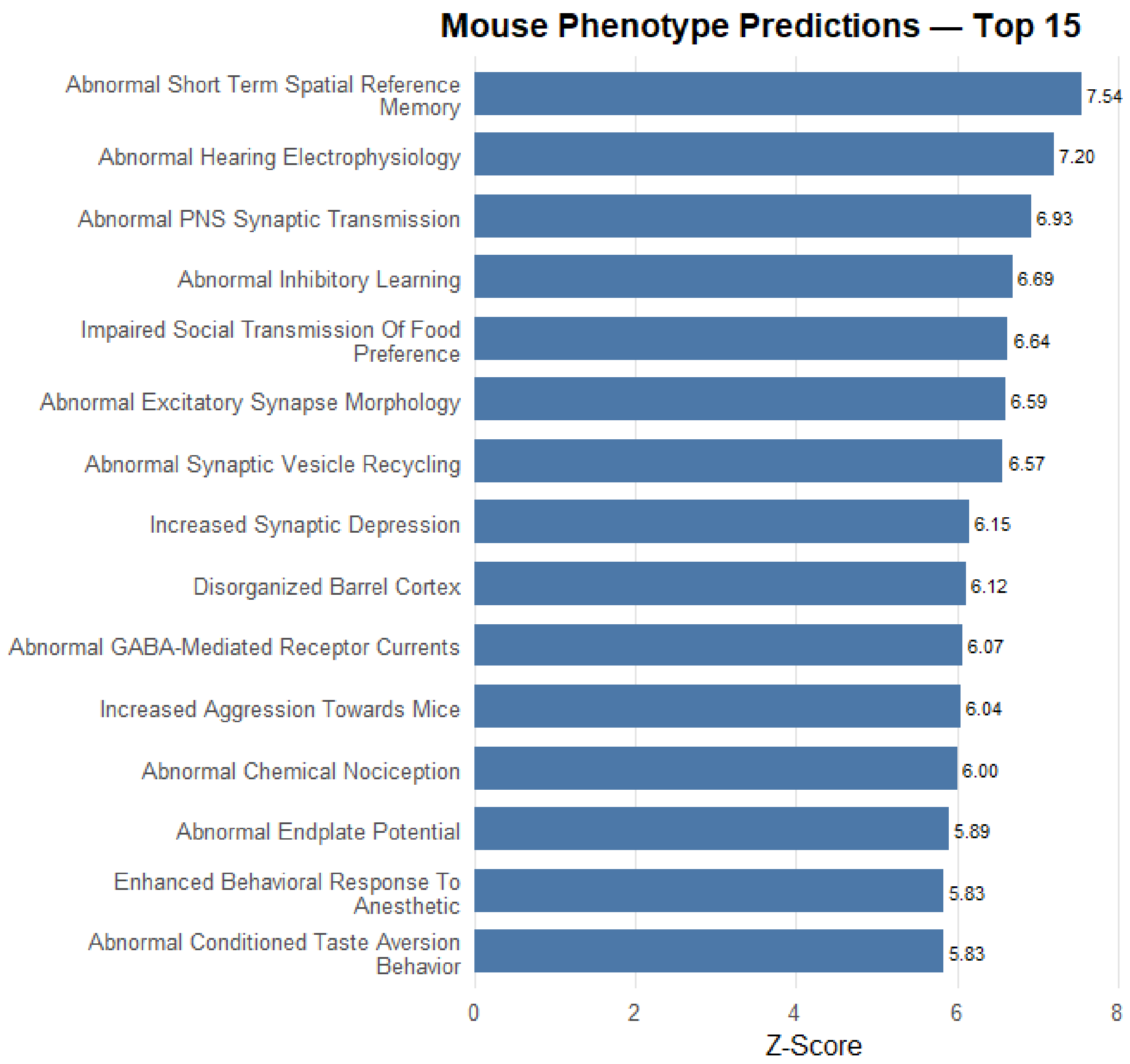

Figure S6. Supplementary Figure S6. lncHUB mouse phenotype predictions for *HTR5A-AS1*. The top phenotype was abnormal short-term spatial reference memory (Z = 7.54), followed by abnormal hearing electrophysiology (Z = 7.20) and abnormal peripheral synaptic transmission (Z = 6.93).

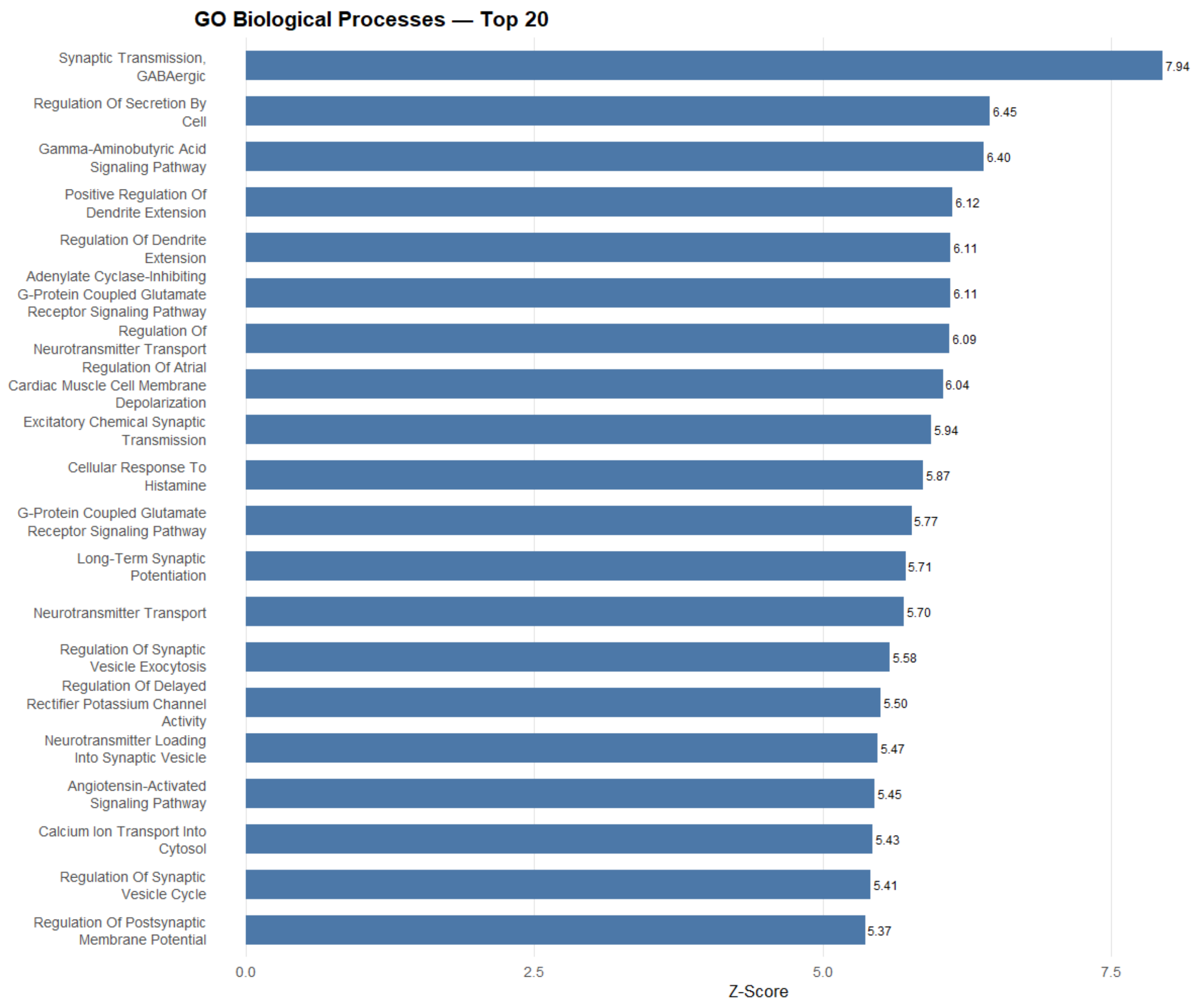

Figure S7. Supplementary Figure S7. Gene Ontology (GO) biological process predictions from lncHUB for *HTR5A-AS1*. The highest-scoring process was GABAergic synaptic transmission (Z = 7.943), followed by regulation of secretion by cell (Z = 6.449) and GABA (Z = 6.396).

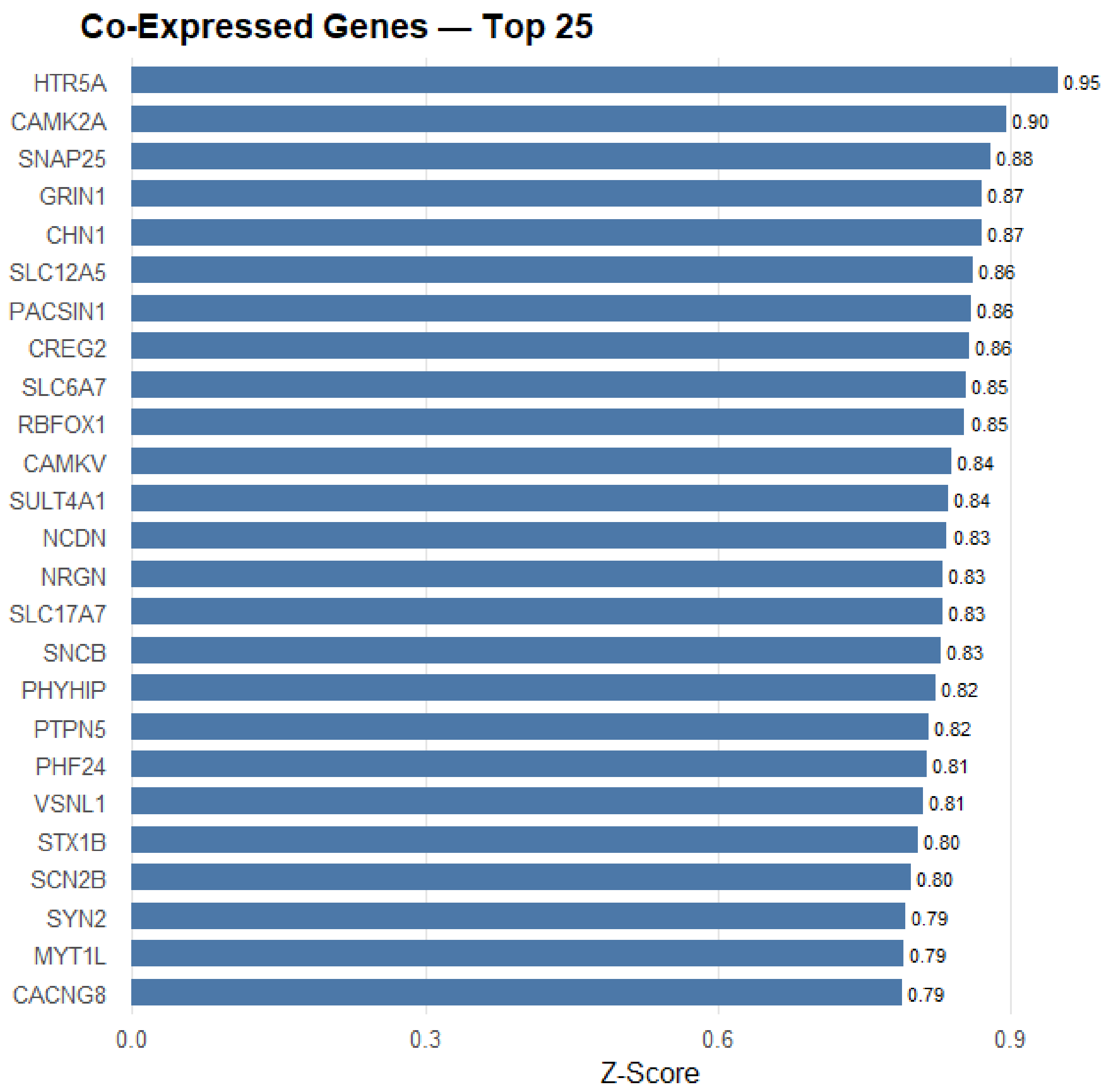

Figure S8. Supplementary Figure S8. Top 25 protein-coding genes co-expressed with *HTR5A-AS1* from lncHUB analysis. The highest-ranking gene was *HTR5A* (Z = 0.948), followed by *CAMK2A* (Z = 0.896), *SNAP25* (Z = 0.880), and *GRIN1* (Z = 0.871).
